# Tetra­kis(4-*tert*-butyl­benz­yl)silane

**DOI:** 10.1107/S1600536810034173

**Published:** 2010-08-28

**Authors:** Lauren E. Burnham, Rulla M. Kachlan, Andy A. Thomas, Craig A. Ogle, Daniel S. Jones

**Affiliations:** a Department of Chemistry, The University of North Carolina at Charlotte, 9201 University City Blvd., Charlotte, NC 28223, USA

## Abstract

The title compound, C_44_H_60_Si, was prepared as an inter­nal standard for diffusion-ordered NMR spectroscopy. The Si atom lies on a special position with 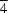 site symmetry.

## Related literature

For applications of the title compound in NMR spectroscopy, see: Li *et al.* (2009 ▶). For similar structures in the same space group, see: Liao *et al.* (2002 ▶); Laliberté *et al.* (2004 ▶). For a previously reported NMR standard, see: Monroe *et al.* (2010 ▶). For a description of the Cambridge Structural Database, see: Allen (2002 ▶).
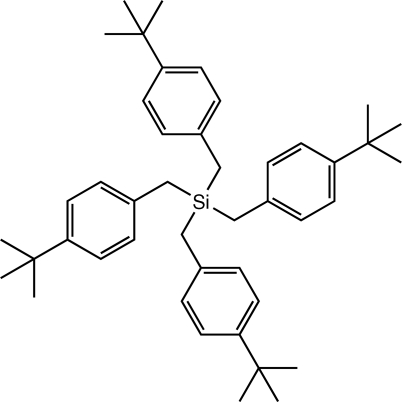


## Experimental

### 

#### Crystal data

C_44_H_60_Si
*M* *_r_* = 617.01
Tetragonal, 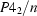

*a* = 17.394 (2) Å
*c* = 6.3613 (6) Å
*V* = 1924.7 (4) Å^3^
*Z* = 2
Cu *K*α radiation
μ = 0.72 mm^−1^
*T* = 295 K
0.31 × 0.15 × 0.12 mm

#### Data collection

Enraf–Nonius CAD-4 diffractometer
5204 measured reflections
1738 independent reflections
1056 reflections with *I* > 2σ(*I*)
*R*_int_ = 0.036
3 standard reflections every 113 reflections  intensity decay: 2%

#### Refinement

*R*[*F* ^2^ > 2σ(*F* ^2^)] = 0.041
*wR*(*F* ^2^) = 0.135
*S* = 1.02
1738 reflections
106 parameters
H-atom parameters constrained
Δρ_max_ = 0.13 e Å^−3^
Δρ_min_ = −0.11 e Å^−3^

### 

Data collection: *CAD-4 EXPRESS* (Enraf–Nonius, 1994 ▶); cell refinement: *CAD-4 EXPRESS*; data reduction: *XCAD4* (Harms & Wocadlo, 1995 ▶); program(s) used to solve structure: *SHELXS97* (Sheldrick, 2008 ▶); program(s) used to refine structure: *SHELXL97* (Sheldrick, 2008 ▶); molecular graphics: *ORTEP-3 for Windows* (Farrugia, 1997 ▶) and *Mercury* (Macrae *et al.*, 2006 ▶); software used to prepare material for publication: *WinGX* (Farrugia, 1999 ▶).

## Supplementary Material

Crystal structure: contains datablocks global, I. DOI: 10.1107/S1600536810034173/su2206sup1.cif

Structure factors: contains datablocks I. DOI: 10.1107/S1600536810034173/su2206Isup2.hkl

Additional supplementary materials:  crystallographic information; 3D view; checkCIF report

## Figures and Tables

**Fig. 1.**
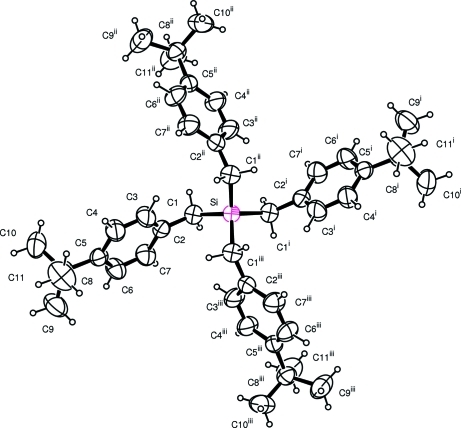
View of title molecule with 50% probability displacement ellipsoids [Symmetry codes: (i) -x + 1/2, -y + 1/2, z (ii) y, -x + 1/2, -z + 1/2 (iii) -y + 1/2, x, -z + 1/2].

**Fig. 2.**
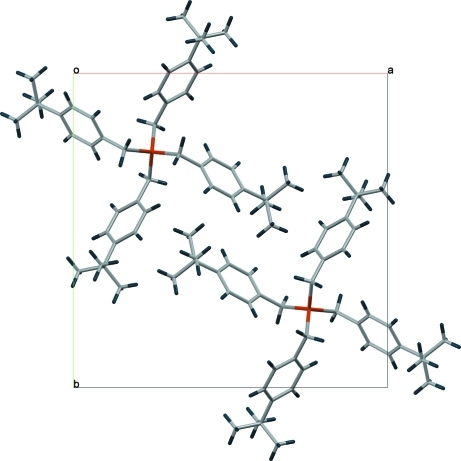
Crystal packing diagram of the title compound viewed along the c axis.

**Fig. 3.**
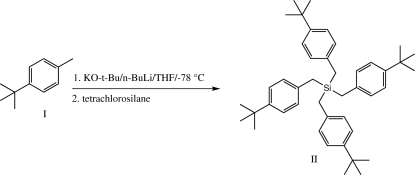
Synthesis scheme.

## supplementary crystallographic information

### Comment

The title compound was prepared as an internal standard for diffusion-ordered NMR spectroscopy. A recent paper on this subject (Li *et al.*, 2009) suggests an internal standard method for correlating diffusion coefficients with formula weights. The title compound was chosen because its shape in solution both approximates that of a spheroid and is similar to that of the species being studied. In addition, it neither reacts with the species under study nor gives interfering NMR signals.

The molecular structure of the title molecule is illustrated in Fig. 1. The molecule sits on a fourfold rotoinversion axis, with the Si atom located at the point of inversion and the four ligands arranged tetrahedrally around the Si atom. The crystal packing of the title compound, viewed along the *c* axis, is illustrated in Fig. 2.

The space group, P4_2_/n, is relatively rare, comprising fewer than 700 of the half-million-plus structures in the Cambridge Structural Database [Version 5.31; Allen, 2002]. Similar structures which crystallized in the same space group include tetrakis(4*-N-t*-Butyl-*N*-aminoxylphenyl)silane (Liao *et al.*, 2002) and tetrakis(4-(Ethoxycarbonylamino)phenyl)silane bis(dioxane) clathrate (Laliberté *et al.*, 2004).

We have previously reported on the crystal structure of another NMR standard of smaller molecular weight, bis(2-naphthylmethyl)diphenylsilane (Monroe *et al.*, 2010).

### Experimental

The synthesis of the title compound is descibed in Fig. 3. A dry, 250 ml Schlenk flask, equipped with a magnetic stirbar, was charged with 4-tertbutyltoluene (I) (7.13 g, 50 mmol), potassium *tert*-butoxide (6.72 g, 55 mmol), then purged with nitrogen. 100 ml of freshly distilled dry THF was added and the reaction was cooled to 195 K. *n*-BuLi (23.91 ml, 2.3*M*, 55 mmol) was then added dropwise. The Schlenk flask was then capped and kept at 233 K overnight. The solution was again cooled to 195 K and 2.09 ml (1.68 g, 10 mmol) of tetrachlorosilane was added dropwise. The reaction mixture was allowed to warm to room temperature and stirred for two hours. The mixture was then quenched with deionized water and extracted three times with petroleum ether. The combined organic layers were dried with magnesium sulfate, filtered, and evaporated. Bulb-to-bulb distillation gave a tan solid, which was recrystallized from petroleum ether to yield pure colorless crystals of tetrakis(4-*tert*-butylbenzyl)silane (II) (3.45 g, 56% recovery).

mp 407–409 K; ^1^H NMR (Toluene-*d_8_*, 300 MHz): 1.27 (*s*)9H, 2.14 (*s*)2H, 6.85 (*d*)2H, 7.20 (*d*)2H. ^13^C NMR (Toluene-*d_8_*, 300 MHz): 31.66, 34.20, 20.71, 124.96, 128.75, 129.52, 147.13. GC/MS (70ev) m/*z*: 469.4, 57.1

### Refinement

The H-atoms were included in calculated positions and treated as riding atoms: C-H = 0.93, 0.97 and 0.96 Å for aromatic CH, CH_2_, and CH_3_ H-atoms, respectively, with U_iso_(H) = k × U_eq_(C), where k = 1.5 for CH_3_ H-atoms, and k = 1.2 for all other H-atoms.

### Crystal data

**Table:**

C_44_H_60_Si	*D*_x_ = 1.065 Mg m^−^^3^
*M**_r_* = 617.01	Cu *K*α radiation, λ = 1.54184 Å
Tetragonal, *P*4_2_/*n*	Cell parameters from 25 reflections
Hall symbol: -P 4bc	θ = 7.8–35.3°
*a* = 17.394 (2) Å	µ = 0.72 mm^−^^1^
*c* = 6.3613 (6) Å	*T* = 295 K
*V* = 1924.7 (4) Å^3^	Prism, colorless
*Z* = 2	0.31 × 0.15 × 0.12 mm
*F*(000) = 676	

### Data collection

**Table:**

Enraf–Nonius CAD-4 diffractometer	θ_max_ = 67.4°, θ_min_ = 3.6°
non–profiled ω/2θ scans	*h* = −20→20
5204 measured reflections	*k* = −20→15
1738 independent reflections	*l* = −7→0
1056 reflections with *I* > 2σ(*I*)	3 standard reflections every 113 reflections
*R*_int_ = 0.036	intensity decay: 2%

### Refinement

**Table:**

Refinement on *F*^2^	H-atom parameters constrained
Least-squares matrix: full	*w* = 1/[σ^2^(*F*_o_^2^) + (0.0761*P*)^2^ + 0.1124*P*] where *P* = (*F*_o_^2^ + 2*F*_c_^2^)/3
*R*[*F*^2^ > 2σ(*F*^2^)] = 0.041	(Δ/σ)_max_ < 0.001
*wR*(*F*^2^) = 0.135	Δρ_max_ = 0.13 e Å^−^^3^
*S* = 1.02	Δρ_min_ = −0.11 e Å^−^^3^
1738 reflections	Extinction correction: *SHELXL97* (Sheldrick, 2008), Fc^*^=kFc[1+0.001xFc^2^λ^3^/sin(2θ)]^-1/4^
106 parameters	Extinction coefficient: 0.0033 (6)
0 restraints	

### Special details

**Table:**

Geometry. All e.s.d.'s (except the e.s.d. in the dihedral angle between two l.s. planes) are estimated using the full covariance matrix. The cell e.s.d.'s are taken into account individually in the estimation of e.s.d.'s in distances, angles and torsion angles; correlations between e.s.d.'s in cell parameters are only used when they are defined by crystal symmetry. An approximate (isotropic) treatment of cell e.s.d.'s is used for estimating e.s.d.'s involving l.s. planes.

### Fractional atomic coordinates and isotropic or equivalent isotropic displacement parameters (Å^2^)

**Table:**

	*x*	*y*	*z*	*U*_iso_*/*U*_eq_	
Si	0.25	0.25	0.25	0.0599 (4)	
C4	0.50538 (12)	0.30509 (12)	0.4293 (4)	0.0780 (7)	
H4	0.5263	0.2856	0.5529	0.094*	
C1	0.33661 (10)	0.25820 (11)	0.0742 (4)	0.0674 (7)	
H1A	0.3507	0.2069	0.0288	0.081*	
H1B	0.3217	0.2868	−0.0502	0.081*	
C5	0.54015 (10)	0.36783 (10)	0.3360 (3)	0.0535 (5)	
C7	0.44050 (11)	0.35854 (12)	0.0714 (4)	0.0668 (6)	
H7	0.4193	0.3779	−0.0519	0.08*	
C8	0.61357 (11)	0.40291 (11)	0.4257 (4)	0.0624 (6)	
C2	0.40700 (10)	0.29575 (10)	0.1652 (4)	0.0580 (5)	
C6	0.50509 (12)	0.39375 (12)	0.1556 (4)	0.0674 (6)	
H6	0.5255	0.4365	0.0876	0.081*	
C3	0.44105 (12)	0.27021 (12)	0.3468 (4)	0.0837 (8)	
H3	0.42	0.2281	0.4163	0.1*	
C9	0.62982 (15)	0.48273 (14)	0.3328 (5)	0.0951 (9)	
H9A	0.634	0.4789	0.1827	0.143*	
H9B	0.6771	0.5023	0.3896	0.143*	
H9C	0.5885	0.517	0.3682	0.143*	
C10	0.68066 (12)	0.35026 (15)	0.3687 (5)	0.0992 (9)	
H10A	0.672	0.3001	0.4269	0.149*	
H10B	0.7275	0.3711	0.4249	0.149*	
H10C	0.6848	0.3465	0.2186	0.149*	
C11	0.60896 (17)	0.41111 (18)	0.6619 (5)	0.1047 (9)	
H11A	0.5674	0.445	0.6977	0.157*	
H11B	0.6564	0.432	0.714	0.157*	
H11C	0.6002	0.3616	0.7241	0.157*	

### Atomic displacement parameters (Å^2^)

**Table:**

	*U*^11^	*U*^22^	*U*^33^	*U*^12^	*U*^13^	*U*^23^
Si	0.0473 (4)	0.0473 (4)	0.0852 (9)	0	0	0
C4	0.0612 (12)	0.0722 (13)	0.101 (2)	−0.0089 (11)	−0.0143 (12)	0.0413 (13)
C1	0.0543 (11)	0.0616 (11)	0.0863 (18)	0.0010 (9)	0.0016 (11)	0.0004 (11)
C5	0.0496 (10)	0.0501 (10)	0.0609 (12)	0.0033 (8)	0.0116 (10)	0.0070 (9)
C7	0.0658 (12)	0.0750 (13)	0.0596 (15)	−0.0063 (10)	0.0042 (10)	0.0141 (11)
C8	0.0584 (11)	0.0644 (12)	0.0645 (15)	−0.0057 (9)	0.0067 (10)	0.0059 (10)
C2	0.0477 (10)	0.0504 (10)	0.0759 (14)	0.0042 (8)	0.0086 (10)	0.0056 (10)
C6	0.0688 (12)	0.0698 (13)	0.0638 (14)	−0.0169 (10)	0.0105 (12)	0.0169 (11)
C3	0.0617 (12)	0.0677 (13)	0.122 (2)	−0.0127 (10)	−0.0093 (14)	0.0472 (14)
C9	0.0946 (17)	0.0790 (15)	0.112 (2)	−0.0315 (13)	−0.0090 (16)	0.0155 (15)
C10	0.0538 (12)	0.111 (2)	0.133 (3)	0.0025 (13)	0.0030 (14)	−0.0136 (19)
C11	0.118 (2)	0.120 (2)	0.0760 (19)	−0.0328 (17)	0.0054 (17)	−0.0061 (17)

### Geometric parameters (Å, °)

**Table:**

Si—C1^i^	1.882 (2)	C8—C11	1.512 (3)
Si—C1^ii^	1.882 (2)	C8—C10	1.527 (3)
Si—C1^iii^	1.882 (2)	C8—C9	1.535 (3)
Si—C1	1.882 (2)	C2—C3	1.372 (3)
C4—C3	1.377 (3)	C6—H6	0.93
C4—C5	1.382 (3)	C3—H3	0.93
C4—H4	0.93	C9—H9A	0.96
C1—C2	1.504 (3)	C9—H9B	0.96
C1—H1A	0.97	C9—H9C	0.96
C1—H1B	0.97	C10—H10A	0.96
C5—C6	1.375 (3)	C10—H10B	0.96
C5—C8	1.526 (3)	C10—H10C	0.96
C7—C2	1.374 (3)	C11—H11A	0.96
C7—C6	1.387 (3)	C11—H11B	0.96
C7—H7	0.93	C11—H11C	0.96
			
C1^i^—Si—C1^ii^	110.68 (7)	C3—C2—C7	116.09 (19)
C1^i^—Si—C1^iii^	107.08 (14)	C3—C2—C1	122.34 (18)
C1^ii^—Si—C1^iii^	110.68 (7)	C7—C2—C1	121.6 (2)
C1^i^—Si—C1	110.68 (7)	C5—C6—C7	122.44 (19)
C1^ii^—Si—C1	107.08 (14)	C5—C6—H6	118.8
C1^iii^—Si—C1	110.68 (7)	C7—C6—H6	118.8
C3—C4—C5	122.7 (2)	C2—C3—C4	121.94 (19)
C3—C4—H4	118.7	C2—C3—H3	119
C5—C4—H4	118.7	C4—C3—H3	119
C2—C1—Si	117.14 (16)	C8—C9—H9A	109.5
C2—C1—H1A	108	C8—C9—H9B	109.5
Si—C1—H1A	108	H9A—C9—H9B	109.5
C2—C1—H1B	108	C8—C9—H9C	109.5
Si—C1—H1B	108	H9A—C9—H9C	109.5
H1A—C1—H1B	107.3	H9B—C9—H9C	109.5
C6—C5—C4	115.05 (19)	C8—C10—H10A	109.5
C6—C5—C8	123.50 (17)	C8—C10—H10B	109.5
C4—C5—C8	121.4 (2)	H10A—C10—H10B	109.5
C2—C7—C6	121.8 (2)	C8—C10—H10C	109.5
C2—C7—H7	119.1	H10A—C10—H10C	109.5
C6—C7—H7	119.1	H10B—C10—H10C	109.5
C11—C8—C5	111.41 (19)	C8—C11—H11A	109.5
C11—C8—C10	109.4 (2)	C8—C11—H11B	109.5
C5—C8—C10	108.13 (17)	H11A—C11—H11B	109.5
C11—C8—C9	107.9 (2)	C8—C11—H11C	109.5
C5—C8—C9	111.83 (18)	H11A—C11—H11C	109.5
C10—C8—C9	108.09 (19)	H11B—C11—H11C	109.5

Symmetry codes: (i) −*y*+1/2, *x*, −*z*+1/2; (ii) −*x*+1/2, −*y*+1/2, *z*; (iii) *y*, −*x*+1/2, −*z*+1/2.
